# Thickness Control of the Spin-Polarized Two-Dimensional Electron Gas in LaAlO_3_/BaTiO_3_ Superlattices

**DOI:** 10.1038/s41598-017-18858-x

**Published:** 2018-01-11

**Authors:** Chen Chen, Le Fang, Jihua Zhang, Guodong Zhao, Wei Ren

**Affiliations:** 1 International Center for Quantum and Molecular Structures, Physics Department, Shanghai University, Shanghai, 200444 China; 2 Materials Genome Institute and Shanghai Key Laboratory of High Temperature Superconductors, Shanghai University, Shanghai, 200444 China; 3 Guizhou Provincial Key Laboratory of Computational Nano-Material Science, Guizhou Education University, Guiyang, 550018 China

## Abstract

We explored the possibility of increasing the interfacial carrier quantum confinement, mobility and conductivity in the (LaAlO_3_)_n_/(BaTiO_3_)_n_ superlattices by thickness regulation using the first-principles electronic structure calculations. Through constructing two different interfacial types of LaAlO_3_/BaTiO_3_ superlattices, we discovered that the LaO/TiO_2_ interface is preferred from cleavage energy consideration. We then studied the electronic characteristics of two-dimensional electron gas (2DEG) produced at the LaO/TiO_2_ interface in the LaAlO_3_/BaTiO_3_ superlattices via spin-polarized density functional theory calculations. The charge carrier density of 2DEG has a magnitude of 10^14^ cm^−2^ (larger than the traditional system LaAlO_3_/SrTiO_3_), which is mainly provided by the interfacial Ti 3d_xy_ orbitals when the thicknesses of LaAlO_3_ and BaTiO_3_ layers are over 4.5 unit cells. We have also revealed the interfacial electronic characteristics of the LaAlO_3_/BaTiO_3_ system, by showing the completely spin-polarized 2DEG mostly confined at the superlattice interface. The interfacial charge carrier mobility and conductivity are found to be converged beyond the critical thickness. Therefore, we can regulate the interfacial confinement for the spin-polarized 2DEG and quantum transport properties in LaAlO_3_/BaTiO_3_ superlattice via controlling the thicknesses of the LaAlO_3_ and BaTiO_3_ layers.

## Introduction

Two-dimensional electron gas (2DEG) can be generated at the interface of heterojunction constructed by two insulating perovskite materials. The 2DEG has many unique electronic properties, such as ferroelectric polarization enhancement^1^, high carrier mobility^2^, two-dimension superconductivity^3^, magnetism at the interface^4^, electronic spin polarization^5,6^, and so on. With the rapid development of thin film growth technology including molecular beam epitaxy^7^, chemical vapor deposition^8^, and pulsed laser deposition^9^, the nature and functionalities of 2DEG draw great attention from both experimental and theoretical points of view.

Generally, there are two primary mechanisms to be involved in the formation of 2DEG at the heterojunction interface: oxygen vacancies^10^ and electronic reconstruction^11^. A prominent experiment is LaAlO_3_/SrTiO_3_ system, in which the LaAlO_3_ slab is grown on the SrTiO_3_ substrate^12^. It is well known that a metallic phase is confined within several monolayers near the LaO/TiO_2_ interface and therefore formed a 2DEG. The LaAlO_3_ slab consists of alternating charged layers LaO and AlO_2_, while the SrTiO_3_ substrate can be considered a stack of alternating neutral layers SrO and TiO_2_ along the (001) direction. The polarization discontinuity at the LaO/TiO_2_ interface causes the electrostatic potential divergence. To avoid the polar discontinuity, half an electron is transferred from the charged layer LaO to the neutral layer TiO_2_^11^, resulting in a metallic phase at the interface of the LaAlO_3_/SrTiO_3_ system.

It is found that the 2DEG may even become magnetic at low temperature by Brinkman *et al*.^13^, as generated at the LaO/TiO_2_ interface between the non-magnetic LaAlO_3_ and SrTiO_3_ materials. This interesting phenomenon has the origin of the induced electrons exchange splitting in the Ti-3d orbital^14^, which is confirmed by the first principles calculations with spin-polarization. Formation of the completely spin-polarized 2DEG is extremely promising for spintronics applications. From the previous work of Nazir *et al*.^15^, LaAlO_3_/BaTiO_3_ model shows higher interfacial charge carrier density, stronger quantum confinement, and larger magnetic moment than the previously reported SrTiO_3_-based model superlattices. In this work, we carry out first principles calculations of the LaAlO_3_/BaTiO_3_ system with a special emphasis of oxide thickness control. As a matter of fact, previous experiments^16^ of LaAlO_3_/BaTiO_3_ superlattices have been realized with 20 periodic numbers and varied stacking periodicity of 2/2 (2 unit cells LaAlO_3_/2 unit cells BaTiO_3_), 3/3, 4/4, 5/5 and 8/8.

BaTiO_3_ is a tetragonal perovskite-type structure at the room temperature, when the temperature is above 393 K, the bulk BaTiO_3_ changes from the tetragonal to cubic structure with a lattice constant of 4.012 Å^17^, band gap of 3.2 eV^18^, and Curie temperature of 415 K^19^. LaAlO_3_ is the other building block for the LaAlO_3_/BaTiO_3_ model and it can be used as substrate for growing many lattice-mismatched perovskite materials. At room temperature, LaAlO_3_ has a perovskite-like structure with a slight rhombohedral distortion, at high temperature LaAlO_3_ also has the cubic perovskite structure with a lattice constant of 3.789 Å, band gap of 5.6 eV^12^, and it has a lattice mismatch of 5.56% with BaTiO_3_ material.

In this work, for the first-principles electronic structure calculations, we first build two kinds of LaAlO_3_/BaTiO_3_ superlattices with different interfaces. For example, in the (LaAlO_3_)_4.5_/(BaTiO_3_)_4.5_ superlattice, we find that the LaO/TiO_2_ interface is the more preferential configuration due to its highest cleavage energy, compared with the BaO/AlO_2_ interface^15,20–23^. Furthermore, we show that the LaAlO_3_/BaTiO_3_ superlattices with LaO/TiO_2_ interface could produce the spin-polarized 2DEG, and the completely spin-polarized 2DEG is strongly confined at the interfacial TiO_2_ layer when the thicknesses of LaAlO_3_ and BaTiO_3_ layers are both larger than 4.5 unit cells.

By means of the analysis of the layer-resolved partial density of states (DOS), we discovered that the mechanism for the spin-polarized 2DEG formation is similar to the LaAlO_3_/SrTiO_3_ systems^11^. Half an electron transfers from the LaO layer to the TiO_2_ layer in the interface of non-stoichiometric LaAlO_3_/BaTiO_3_ superlattices, in which two symmetrical and identical LaO/TiO_2_ interfaces are considered in the computation^24^. The spin-polarized 2DEG contributed by the interfacial Ti 3d_xy_ orbitals is found to be tightly confined at the interfacial TiO_2_ monolayer, corresponding to the theoretically computed charge carrier density of 2.25 × 10^14^ cm^−2^ in LaAlO_3_/BaTiO_3_ superlattice when the thicknesses of LaAlO_3_ and BaTiO_3_ are both greater than 4.5 unit cells. Whereas we constructed the superlattice (SrTiO_3_)_4.5_/(LaAlO_3_)_4.5_, the calculated two-dimensional electron gas density at the interface is 1.48 × 10^14^ cm^−2^ (see Fig. S7 in Supplemental Information) which is significantly lower than our LaAlO_3_/BaTiO_3_ superlattice. In order to qualitatively analyze the effect of the thicknesses of LaAlO_3_ and BaTiO_3_ layers on the properties of interfacial transport characteristics, we calculated the relative interfacial charge carrier mobility and conductivity by using the (LaAlO_3_)_2.5_/(BaTiO_3_)_2.5_ superlattice as reference. Our results suggest that, when the thicknesses of LaAlO_3_ and BaTiO_3_ layers become larger than 4.5 unit cells, the mobility and conductivity are converged to the above-mentioned value. Therefore, our work gives theoretical guidance for generating superior spin-polarized 2DEG in LaAlO_3_/BaTiO_3_ superlattice, and enhancing the interfacial transport properties of the charge carrier through adjusting the thicknesses of LaAlO_3_ and BaTiO_3_ layers.

We also consider carefully the effect of ferroelectric BaTiO_3_ on the BaTiO_3_/LaAlO_3_ superlattices. In detail, we constructed tetragonal BaTiO_3_ with lattice constants a = b = 3.9945 Å and c = 4.0335 Å for the different (LaAlO_3_)_n_/(BaTiO_3_)_n_ superlattices (n = 2.5 to 8.5). Note that after relaxation the initial uniaxial ferroelectric structure transforms to two opposite polar modes pointing towards the bulk inside BaTiO_3_ (see Fig. S1 in Supplemental Information), and a tiny residual polarization of about 2.9 µC/cm^2^ which points toward the direction of initial ferroelectric polarization we set in the perovskite BaTiO_3_. This means that our mirror symmetry is not exact and the superlattice presents a pseudo-ferroelectricity. In order to avoid the energy-consuming charged domain wall in the middle of BaTiO_3_ the polarization diminishes (see Fig. S1). Two separate bands are found near the conduction band minima which were actually almost degenerate in the exact mirror-symmetric BaTiO_3_ case (see Fig. S2 in Supplemental Information).

## Results

### Interface energetics

**Figure 1:**
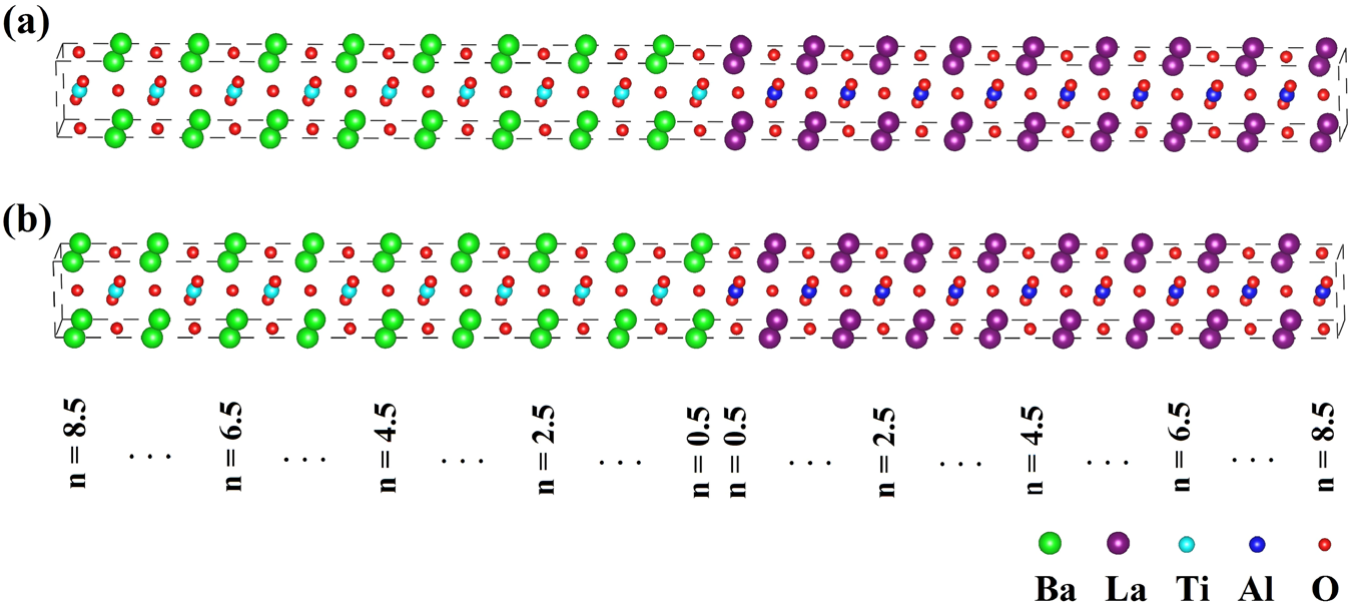
Atomic structures of the supercells with the (**a**) LaO/TiO_2_, (**b**) BaO/AlO_2_ interfaces in (LaAlO_3_)_8.5_/(BaTiO_3_)_8.5_ superlattice.

Periodic boundary conditions are applied when constructing (LaAlO_3_)_n_/(BaTiO_3_)_n_ superlattices along (001) direction^25^, where n is the number of unit cells of LaAlO_3_ and BaTiO_3_ (n = 2.5 to 8.5)^26,27^. Both non-stoichiometric LaAlO_3_ and BaTiO_3_ slabs have two distinct kinds of terminal surfaces^28^, the LaAlO_3_ slab has LaO and AlO_2_ terminal surface, while the BaTiO_3_ substrate has BaO and TiO_2_ terminal surface. Thus, we build two kinds of symmetric interface for (LaAlO_3_)_n_/(BaTiO_3_)_n_ superlattices, including LaO/TiO_2_ or BaO/AlO_2_ interfaces, as shown in Fig. 1. In the supercell we have two interfaces, namely the left/right side interface and the middle interface, respectively. The index n indicates the LaAlO_3_ (BaTiO_3_) unit cells counting from the middle interface location.

In order to compare the interface stability for these two types of interfaces, we introduced the cleavage energy^15,20–23^ E_cleav_ which is defined as:

1 $${{\rm{E}}}_{{\rm{cleav}}}=\,({{\rm{E}}}_{{\rm{LAO}}}+{{\rm{E}}}_{{\rm{BTO}}}-{{\rm{E}}}_{S})/{\rm{2A}}$$

where E_S_ is the total energy for the superlattices containing two kinds of perovskite compositions that are LaAlO_3_ and BaTiO_3_; E_LAO_ and E_BTO_ represent the energy of LaAlO_3_ and BaTiO_3_ slab systems, which have the same superlattice lattice constant with LaAlO_3_/BaTiO_3_ supercell, but BaTiO_3_ or LaAlO_3_ slabs are replaced by the vacuum of same size. The area of an interface in the superlattices is defined as A, and the factor of 2 in the formula indicates two symmetric interfaces in the superlattice models. The physical meaning of cleavage energy is the energy needed to decompose the (LaAlO_3_)_n_/(BaTiO_3_)_n_ superlattices into two sections, and thus, the numerical value of the cleavage energy determines the compactness of the interfacial cohesion between the LaAlO_3_ and BaTiO_3_ slabs, which can describe the thermodynamic stability of the interface^21,22,29^.

We have calculated cleavage energy E_cleav_ for (LaAlO_3_)_n_/(BaTiO_3_)_n_ superlattices (n = 2.5 to 8.5) with LaO/TiO_2_ and BaO/AlO_2_ interfaces, as listed in Table 1. Overall, it can be found that cleavage energy of LaO/TiO_2_ interface is higher than BaO/AlO_2_ interface for the same index n. This means that the superlattices with LaO/TiO_2_ interface are generally more stable than BaO/AlO_2_ interface. With the increase of the parameter n, it is clearly noted that cleavage energy decreased from 0.117 eV/Å^2^ to 0.066 eV/Å^2^ for BaO/AlO_2_ interface and decreased from 0.189 eV/Å^2^ to 0.127 eV/Å^2^ for LaO/TiO_2_ interface. This suggests that with the increase of the superlattice size, the interface becomes less stable.

**Table 1:** Calculated cleavage energy (eV/Å^2^) for the (LaAlO_3_)_n_/(BaTiO_3_)_n_ superlattices (n = 2.5 to 8.5) with the LaO/TiO_2_ and BaO/AlO_2_ interfaces.

Systems	Cleavage energy (eV/Å^2^)	
	LaO/TiO_2_	BaO/AlO_2_
(LaAlO_3_)_2.5_/(BaTiO_3_)_2.5_	0.189	0.117
(LaAlO_3_)_3.5_/(BaTiO_3_)_3.5_	0.175	0.109
(LaAlO_3_)_4.5_/(BaTiO_3_)_4.5_	0.164	0.101
(LaAlO_3_)_5.5_/(BaTiO_3_)_5.5_	0.155	0.092
(LaAlO_3_)_6.5_/(BaTiO_3_)_6.5_	0.147	0.083
(LaAlO_3_)_7.5_/(BaTiO_3_)_7.5_	0.138	0.074
(LaAlO_3_)_8.5_/(BaTiO_3_)_8.5_	0.127	0.066

### 2DEG in LaAlO_3_/BaTiO_3_ superlattices

**Figure 2:**
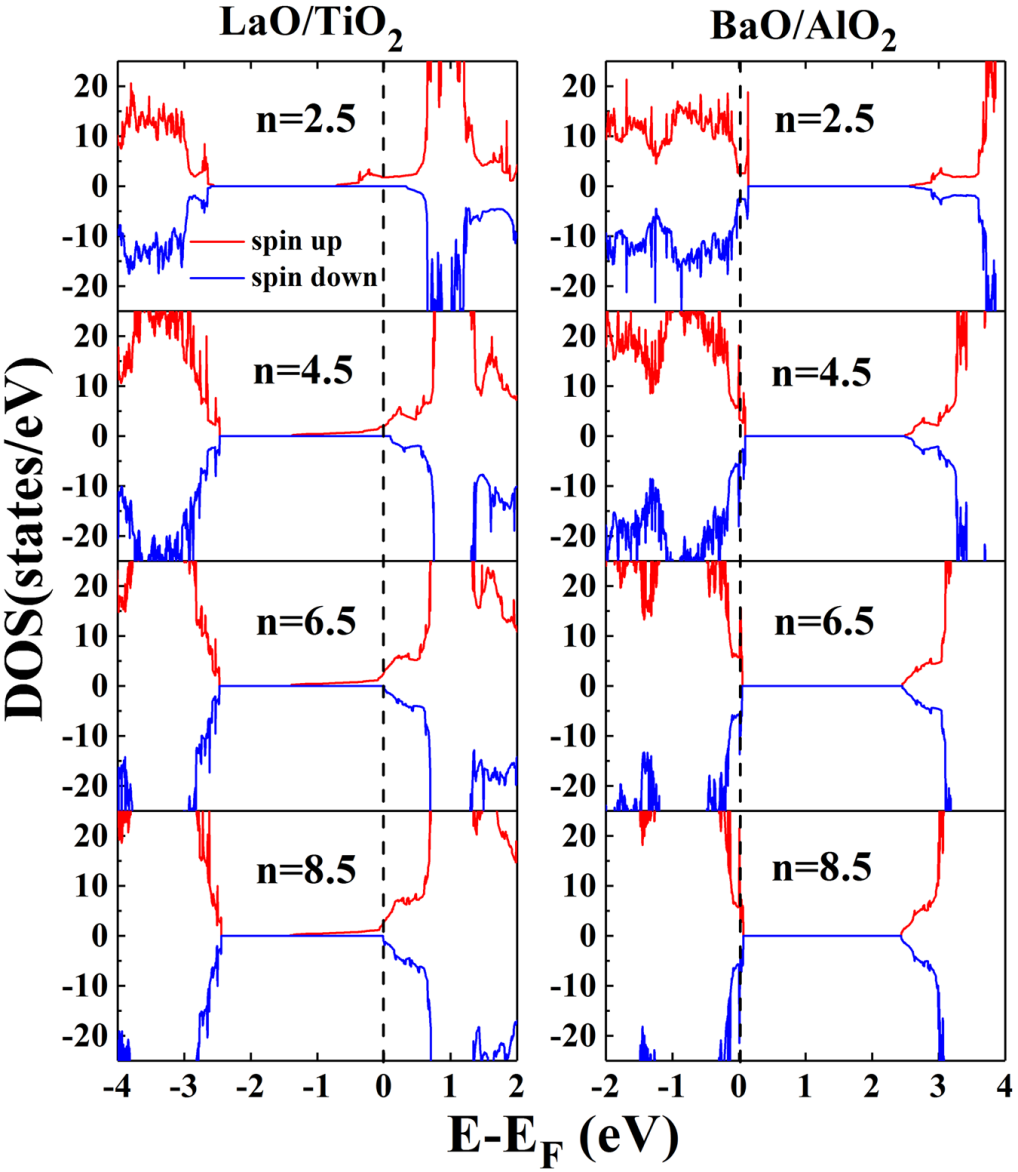
Calculated spin-resolved total density of states (DOS) for the (LaAlO_3_)_n_/(BaTiO_3_)_n_ superlattices (n = 2.5, 4.5, 6.5, and 8.5) with the LaO/TiO_2_ (left column) and BaO/AlO_2_ (right column) interfaces. The red and blue lines show the majority-spin and minority-spin, respectively. The vertical lines at 0 eV indicate the Fermi level in all DOS plots.

To investigate the interface type induced electronic properties modification for the (LaAlO_3_)_n_/(BaTiO_3_)_n_ superlattices, we calculated the total DOS for the LaO/TiO_2_ and BaO/AlO_2_ interfaces for (LaAlO_3_)_n_/(BaTiO_3_)_n_ superlattices (n = 2.5, 4.5, 6.5, and 8.5), as shown in Fig. 2. It can be seen that spin-polarized 2DEG is generated in the superlattices with LaO/TiO_2_ interface. Like the classical model LaAlO_3_/SrTiO_3_, spin-polarized 2DEG is seen at the LaO/TiO_2_ interface, as reported by Nazir *et al*.^30^. Making 2DEG spin-polarized is a very important for spintronics applications, where the regulation the atomic layer types at the interface enlarges the spectrum of potential applications. Experimental scientists can control the interface types by inserting atomic layers, using feasible and widely applied techniques. For example, Yajima *et al*. successfully controlled the SrRuO_3_/Nb:SrTiO_3_ Schottky heterojunction by inserting an artificial interface dipole^31^, and Chen *et al*. enhanced the mobility of 2DEG through inserting a single-unit-cell layer of La_1−x_Sr_x_MnO_3_ at the interface^32^. Inspection of Fig. 2 indicated that the electronic states in the model LaO/TiO_2_ are half-metallic and do not change much in the energy range from −1.4 eV to Fermi level when the index n ≥ 4.5.

**Figure 3:**
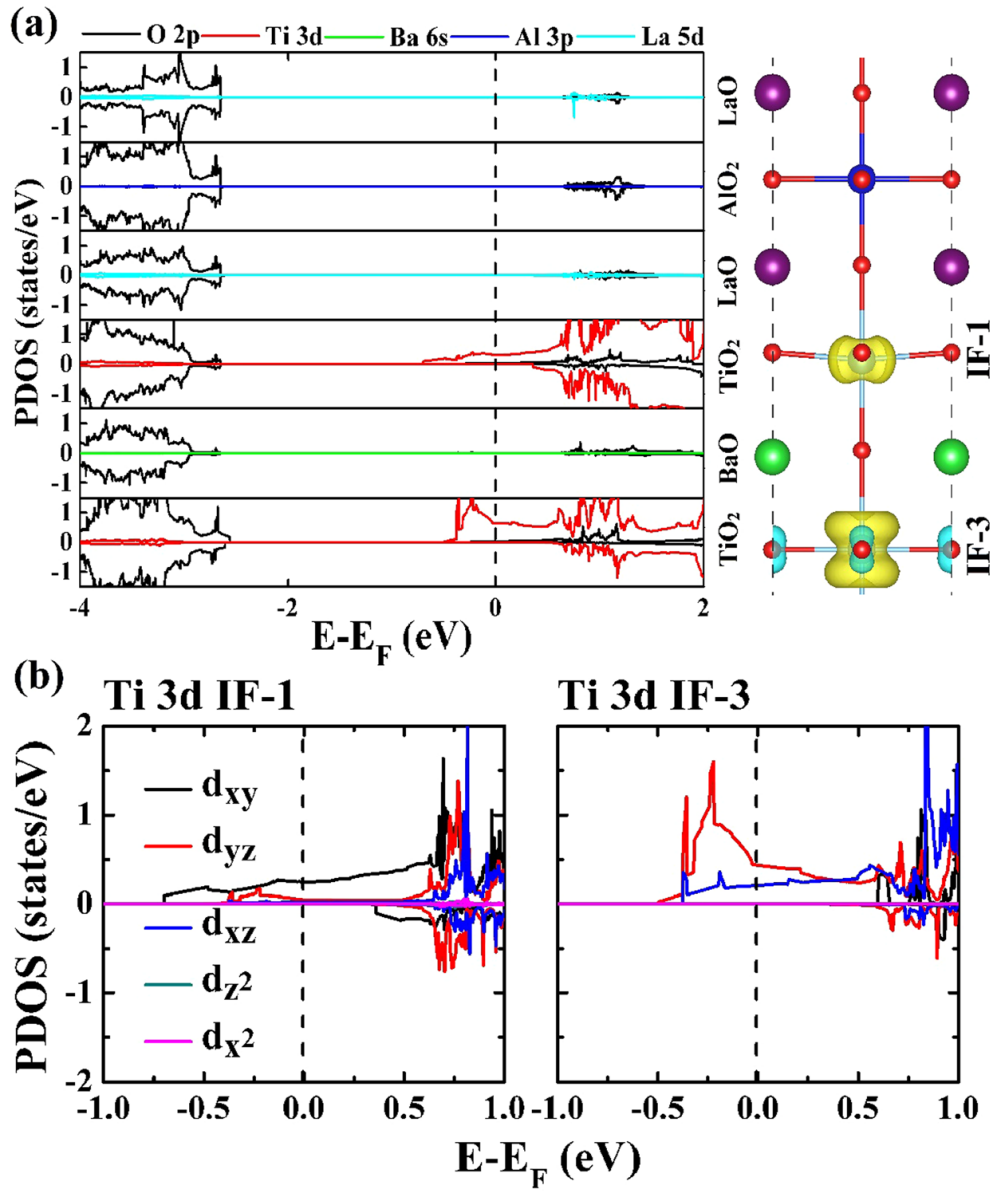
(**a**) Calculated layer-resolved partial DOS for the (LaAlO_3_)_2.5_/(BaTiO_3_)_2.5_ superlattice in the range from −4.0 to 2.0 eV, with the spin density corresponding to each of the oxide monolayers. (**b**) The orbital-resolved partial DOS for Ti atom at the first (IF-1) and the third (IF-3) TiO_2_ monolayers.

It is important to reveal the origin of spin-polarized 2DEG in superlattices (n = 2.5 to 8.5), we thus plot the layer-resolved partial DOS along with the spin charge density for (LaAlO_3_)_2.5_/(BaTiO_3_)_2.5_ superlattice, as shown in Fig. 3(a). We use IF-1, IF-3, IF-5, and IF-7 to represent the first, third, fifth, and seventh TiO_2_ monolayers. The 2DEG originates from Ti 3d states in the IF-1 and IF-3 TiO_2_ monolayers, while the LaAlO_3_ slab does not contribute to the 2DEG in the (LaAlO_3_)_2.5_/(BaTiO_3_)_2.5_ superlattice, demonstrating that the electrons provided by LaO monolayer are shared by Ti atoms in all TiO_2_ layers. In other words, the spin-polarized 2DEG may diffuse from the interface to the inside of the BaTiO_3_. The orbital-resolved partial DOS figures, as shown in Fig. 3(b), suggest that the half-metallic states at IF-1 TiO_2_ monolayer are mostly from Ti 3d_xy_ orbitals, Ti 3d_yz_ orbitals and Ti 3d_xz_ orbitals only give a little contribution. While at IF-3 TiO_2_ monolayer, different from IF-1, most of the half-metallic states come from Ti 3d_yz_ orbitals, partially from Ti 3d_xz_ orbital and none from Ti 3d_xy_ orbital.

**Figure 4:**
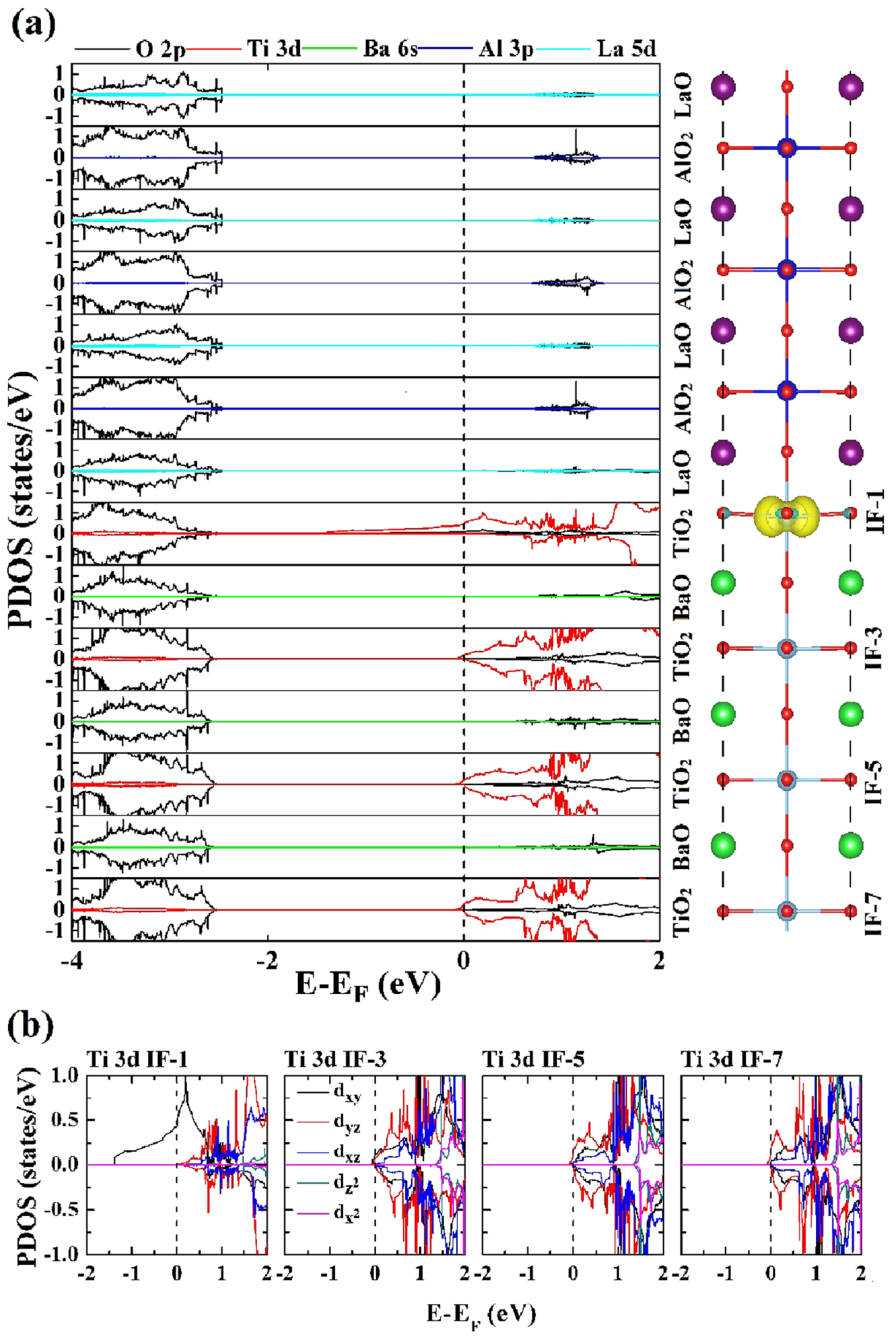
(**a**) Calculated layer-resolved partial DOS for (LaAlO_3_)_6.5_/(BaTiO_3_)_6.5_ model in the range from −4.0 to 2.0 eV, together with the spin density projected on to each of the oxide monolayers. (**b**) The orbital-resolved DOS for Ti atom at the first (IF-1), third (IF-3), fifth (IF-5), and seventh (IF-7) TiO_2_ monolayers, respectively.

Now let us look at the distribution of the 2DEG and the contribution of Ti atomic orbitals as the thickness parameter n increases, hence the layer-resolved partial DOS along with the spin charge density for (LaAlO_3_)_6.5_/(BaTiO_3_)_6.5_ superlattice is shown in Fig. 4(a). It is noted that nearly all the half-metallic states are contributed by the interfacial TiO_2_ monolayer, and the contributions from the IF-3, IF-5, and IF-7 TiO_2_ layer are negligible. This clearly indicates that the spin-polarized 2DEG is tightly confined at the interfacial TiO_2_ layer.

The mechanism for 2DEG formation is similar to the LaAlO_3_/SrTiO_3_ systems, in the (LaAlO_3_)_n_/(BaTiO_3_)_n_ superlattices (n = 2.5 to 8.5) with LaO/TiO_2_ interface, an “extra” electron is injected into the supperlattice as a result of an electron located at the additional LaO layer^24^. In order to analyze the contribution of the Ti 3d orbitals to the 2DEG, we plot the orbital-resolved partial DOS in Fig. 4(b). The spin-polarized 2DEG located at IF-1 TiO_2_ monolayer is entirely contributed by Ti 3d_xy_ orbitals, and there is no obvious 2DEG in other TiO_2_ monolayers near the Fermi level. Briefly, we find that the spin-polarized 2DEG is tightly confined at the interfacial TiO_2_ monolayer in (LaAlO_3_)_6.5_/(BaTiO_3_)_6.5_ superlattice, and completely provided by the interfacial Ti 3d_xy_ orbitals. Through a comparison of the superlattices with thickness n = 2.5 and n = 6.5, the results show that the electronic property has a strong dependence on the superlattice size. Such tightly confined 2DEG at the interfacial TiO_2_ monolayer, contributed by the Ti 3d states, is very consistent with the results of Nazir *et al*.^15^. Moreover, we have also find the difference of Ti 3d orbitals contribution to the DOS located near the interface when the period of superlattice is as short as n = 2.5.

### Interfacial charge carrier density

**Figure 5:**
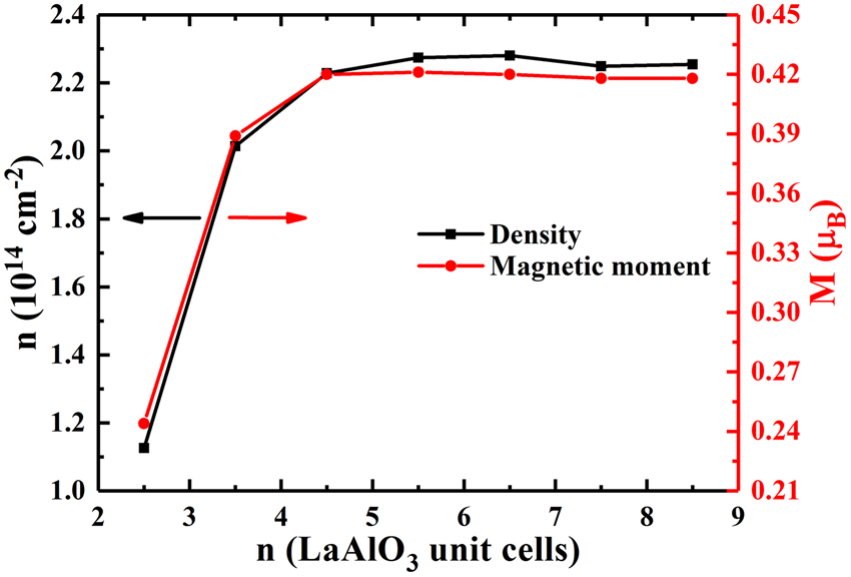
Calculated interfacial charge carrier density for (LaAlO_3_)_n_/(BaTiO_3_)_n_ superlattices (n = 2.5 to 8.5) with LaO/TiO_2_ interface and the magnetic moment of the Ti atom at the interface as a function of the index n.

Next, we calculated the interfacial charge carrier density^21,28,33,34^ for the superlattices via integrating the partial DOS near the Fermi level of the occupied Ti 3d orbitals located at the interfacial TiO_2_ monolayer^21,29^, and displayed the relation between the interfacial charge carrier density and the parameter n, as shown in Fig. 5 (black line). When n ≤ 4.5, the interfacial charge carrier density increased from 1.13 × 10^14^ cm^−2^ to 2.25 × 10^14^ cm^−2^ with the increase of the index n, when n ≥ 4.5, the interfacial charge carrier density was converged to 2.25 × 10^14^ cm^−2^. From the work of Meevasana *et al*.^35^, the 2DEG with the electron density was measured to be as large as 8 × 10^13^ cm^−2^, formed at the bare SrTiO_3_ surface in LaAlO_3_/SrTiO_3_ system. We also constructed the (SrTiO_3_)_4.5_/(LaAlO_3_)_4.5_ superlattice and found the 2DEG density at the interface is 1.48 × 10^14^ cm^−2^. We plot the layer-resolved partial DOS for (SrTiO_3_)_4.5_/(LaAlO_3_)_4.5_ superlattice, as shown in the Fig. S7. By comparison, the system we studied can form a 2DEG with even higher charge carrier density than the traditional LaAlO_3_/SrTiO_3_ systems^15^. For LaAlO_3_/SrTiO_3_ superlattice we found that there is more electron density penetrating into the SrTiO_3_ near the interface. This explains why we could obtain a higher electron density of LaAlO_3_/BaTiO_3_ superlattice than that of LaAlO_3_/SrTiO_3_ superlattice.

In order to ensure the reliability of the relationship between interfacial charge carrier density and the parameter n, we also calculated local magnetic moment^29,33,36^ on Ti atoms of the interfacial TiO_2_ monolayer. From Fig. 5 (red line), we observe that the magnetic moment versus n shows a similar trend with the relationship between the interfacial charge carrier density and the parameter n. The Ti atomic magnetic moment increased from 0.24 µ_B_ to 0.42 µ_B_ with the increase of the parameter n, and then converged to 0.42 µ_B_ when the thicknesses of LaAlO_3_ and BaTiO_3_ layers are greater than 4.5 unit cells. Similar to LaAlO_3_/SrTiO_3_ systems, for the (LaAlO_3_)_n_/(BaTiO_3_)_n_ superlattices, about half electron is transferred from addition LaO monolayer to the neighboring TiO_2_ monolayer in the interface as a result of the polar discontinuity, the half-electron will occupy partial Ti 3d orbitals^37^, and our saturation value result is very close to 0.46 µ_B_ reported by Nazir *et al*.^15^.

### Calculation of electron effective mass

**Figure 6:**
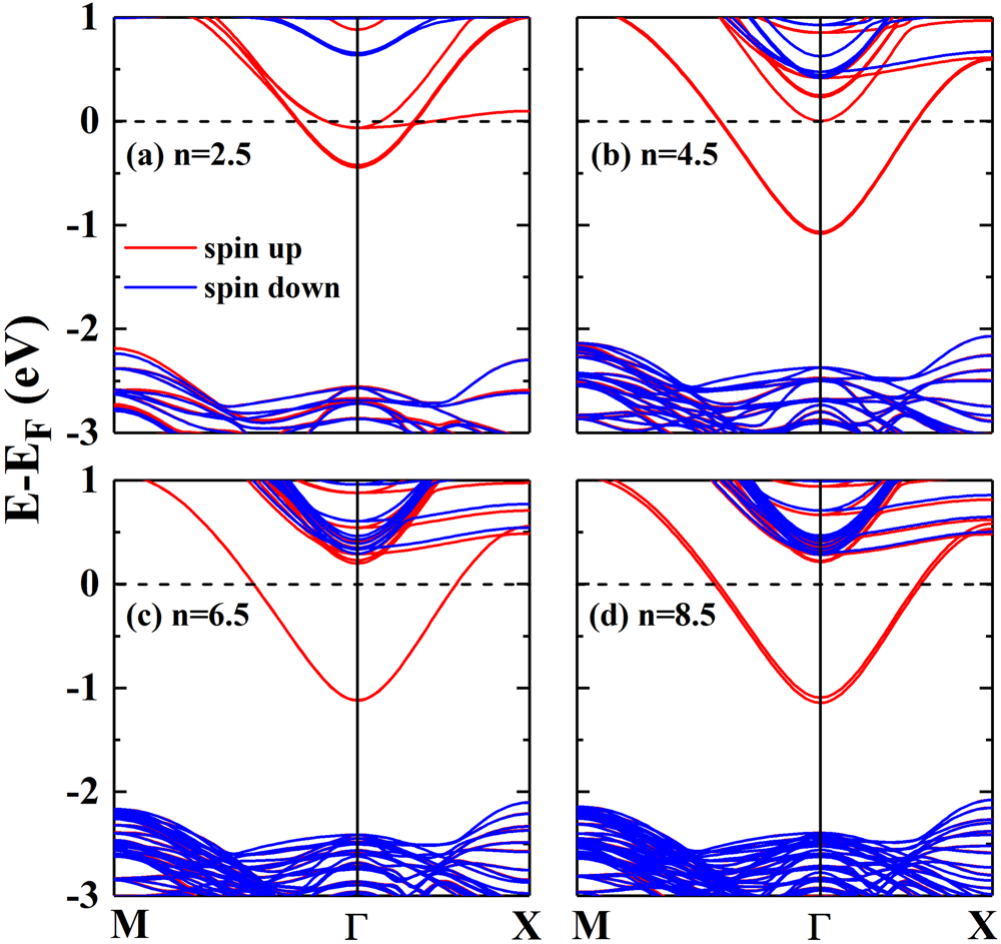
Electronic band structures for the (**a**) n = 2.5, (**b**) n = 4.5, (**c**) n = 6.5, and (**d**) n = 8.5 (LaAlO_3_)_n_/(BaTiO_3_)_n_ superlattices, respectively. The red (blue) lines show the majority (minority)-spin sates, and the black horizontal dashed line at 0 eV indicates the Fermi level. The bottom conduction band is employed to calculate the relative electron effective mass.

To further discuss the effect of superlattice size on electronic properties of the 2DEG, we calculated the band structure along the path M-Г-X of the Brillouin zone^21,22,38^, as shown in Fig. 6. We can see that there is only majority-spin electrons pass through the Fermi level, resulting in the spin-polarized 2DEG at the superlattice interface. Figure 6 also suggested that the bottom conduction band becomes deeper in energy as the parameter n increases. Moreover, such band consists two degenerate energy levels and splits into two bands when n = 8.5. The degenerate states are due to the fact that there are two identical interfaces in the superlattice, whereas a slight symmetry breaking in the superlattice structure may result in the degenerate level splitting. Through the above DOS analysis, we realize that the conduction band passing through the Fermi level is provided by the Ti 3d orbitals, mainly supplied by Ti 3d_xy_ state.

It is important to qualitatively compare the interfacial electron transport characteristics in the (LaAlO_3_)_n_/(BaTiO_3_)_n_ superlattices. We calculated the electron relative effective masses (m*/m_e_) along the Г-X and Г-M directions using the bottom conduction band for each superlattice, in which m_e_ is the electron mass, and the results are listed in Table 2. The calculated electron effective masses are obtained from the following formula^20–22,38^:

2 $$\frac{1}{{{\rm{m}}}^{\ast }}=\frac{1}{{{\rm{\hbar }}}^{2}}\,\frac{{\partial }^{2}{{\rm{E}}}_{{\rm{CB}}}}{\partial {{\rm{k}}}^{2}}$$

where ħ is the reduced Planck constant, E_CB_ and k are the energy and wavevector of the bottom conduction band, respectively. From the Table 2 we can clearly find that the relative effective masses, whether along the Г-X or Г-M direction, decreased from 0.351 to 0.273 with the increase of the parameter n. When n ≥ 4.5, the m*/m_e_ is converged to 0.273, suggesting that the relative effective mass of the 2DEG becomes a constant. We can also find almost no difference between the two kinds of relative effective mass, indicating same characteristics along the two transport directions for the 2DEG. In the typical LaAlO_3_/SrTiO_3_ systems, the relative effective mass of the 2DEG confined at SrTiO_3_ surface is 0.5–0.6 measured by the angle-resolved photoemission spectroscopy from the report of Meevasana *et al*.^35^. Therefore our studied 2DEG has a smaller effective mass, and thus better carrier mobility than the LaAlO_3_/SrTiO_3_ systems.

**Table 2:** Calculated relative electron effective mass m*/m_e_ along the Г-X and Г-M paths for the (LaAlO_3_)_n_/(BaTiO_3_)_n_ superlattices.

Systems	m*/m_e_	
	Г-X	Г-M
(LaAlO_3_)_2.5_/(BaTiO_3_)_2.5_	0.351	0.351
(LaAlO_3_)_3.5_/(BaTiO_3_)_3.5_	0.285	0.285
(LaAlO_3_)_4.5_/(BaTiO_3_)_4.5_	0.273	0.273
(LaAlO_3_)_5.5_/(BaTiO_3_)_5.5_	0.273	0.274
(LaAlO_3_)_6.5_/(BaTiO_3_)_6.5_	0.273	0.274
(LaAlO_3_)_7.5_/(BaTiO_3_)_7.5_	0.274	0.275
(LaAlO_3_)_8.5_/(BaTiO_3_)_8.5_	0.274	0.274

When considering the ferroelectricity of BaTiO_3_ in (LaAlO_3_)_n_/(BaTiO_3_)_n_ superlattices (n = 2.5, 4.5, 6.5, and 8.5), by observing the change of its properties, two separate bands are found near the conduction band minima which were actually almost degenerate in the exact mirror-symmetric BaTiO_3_ case (see Figs S2–4 in Supplemental Information and Fig. 6). This shows that the two interfaces will exhibit different electronic band structure characteristics when BaTiO_3_ is in ferroelectric state.

### Charge carrier mobility and conductivity

**Figure 7:**
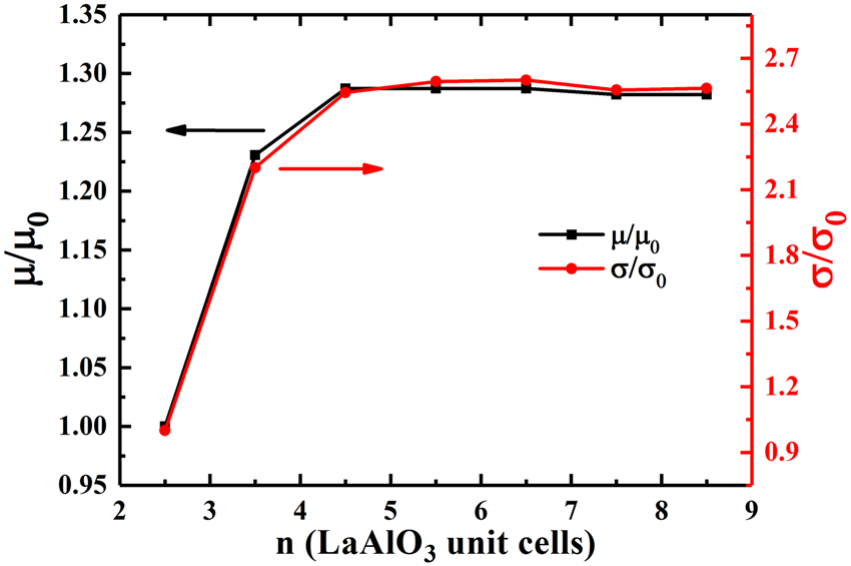
Calculated relative interfacial electron mobility (µ/µ_0_) and electrical conductivity (σ/σ_0_) for the (LaAlO_3_)_n_/(BaTiO_3_)_n_ superlattices, where the µ_0_ and σ_0_ representative the interfacial electron mobility and electrical conductivity for the (LaAlO_3_)_2.5_/(BaTiO_3_)_2.5_ reference system, respectively.

Now let us compare the interfacial electron mobility and conductivity in the (LaAlO_3_)_n_/(BaTiO_3_)_n_ superlattices. For that, we calculated the relative interfacial electron mobility (µ/µ_0_) and electrical conductivity (σ/σ_0_)^21,22,38,39^ with respect to (LaAlO_3_)_2.5_/(BaTiO_3_)_2.5_ superlattice, and plotted the results as function of the index n, as shown in Fig. 7. We employed the following formulas:

3 $$\mu \,={\rm{e}}\langle{\rm{\tau }}\rangle/{{\rm{m}}}^{\ast }$$

4 $${\rm{\sigma }}\,=\mathrm{ne}\mu $$

where e, ‹τ›, m*, n, µ, and σ represent elemental charge, average scattering time, effective mass, charge carrier density, carrier mobility, and electrical conductivity, respectively. The scattering time ‹τ› is assumed to be a constant^40^, determined by several scattering factors such as phonon scattering, electron-electron scattering, impurity scattering, and so on^41,42^. Our results show that, when the thicknesses of LaAlO_3_ and BaTiO_3_ layers are greater than 4.5 unit cells, the superlattices have a high and saturated carrier mobility and conductivity. Interestingly, the carrier conductivity for superlattices with n ≥ 4.5 is about 2.6 times than the reference superlattice, which is consistent with the results of the interfacial charge carrier density and the relative charge carrier effective mass. These results show that we can obtain a spin-polarized 2DEG by regulating the thicknesses of LaAlO_3_ and BaTiO_3_ layers in the process of growing LaAlO_3_/BaTiO_3_ superlattices.

## Conclusion

In summary, spin-polarized density functional theory calculations are employed to investigate the possibility of enhancing the 2DEG quantum confinement at LaO/TiO_2_ interface and the interfacial charge carrier density, mobility, and conductivity in LaAlO_3_/BaTiO_3_ superlattice by using thickness control. We find that the superlattices with LaO/TiO_2_ interface are more stable than BaO/AlO_2_ from the cleavage energy calculations. When the critical thicknesses of LaAlO_3_ and BaTiO_3_ layers are over 4.5 unit cells, the spin-polarized 2DEG is mainly provided by the interfacial Ti 3d_xy_ orbitals and completely confined at the interface. In such cases, through the study of the interfacial charge carrier density, mobility, and conductivity, we confirm that these properties become independent of the thickness. Our present work provides theoretical understanding to produce and enhance the interfacial quantum confinement for spin-polarized 2DEG and the interfacial transport properties by adjusting the thicknesses of LaAlO_3_ and BaTiO_3_ layers. In LaAlO_3_/BaTiO_3_ superlattice, electrons are almost bound at the interfaces, but for LaAlO_3_/SrTiO_3_ superlattice we found that there is electron density penetrating into the SrTiO_3_ near the interface.

### Computational and Structural Details

In this study, for all density functional theory (DFT)^43^ calculations we used Vienna Ab initio Simulation Package(VASP)^44^. The electron exchange-correlation interaction was described by the generalized gradient approximation (GGA) parametrized by the Perdew-Burke-Ernzerhof (PBE) plus the on-site Coulomb interaction approach (GGA + U)^45^. The projected augmented method (PAW) was used to describe the electron-ion interaction. In order to more accurately describe the electronic states of strongly correlated electrons, effective U value of 5.8 eV and 7.5 eV were applied to Ti 3d and La 4 f orbitals^46,47^, respectively. Such U values are well tested and more results are in Figs S5–6 of the Supplemental Information. Our calculations were performed by employing a cutoff energy of 450 eV for the plane wave basis set and a Monkhorst-Pack k-point mesh of 6 × 6 × 1 (except for n = 2.5 we use 6 × 6 × 2). The convergence of electronic energy was set to 10^−6^ eV in the self-consistent calculations. The atomic positions were optimized to simulate the epitaxial superlattice until the interatomic forces were small than 0.03 eV/Å.

We used slab model to build (LaAlO_3_)_n_/(BaTiO_3_)_n_ superlattices (n = 2.5 to 8.5) by staking the epitaxial slabs using BaTiO_3_ lattice parameters, in which n is a half-integer representing the number of LaAlO_3_ and BaTiO_3_ unit cells along (001) direction. We have constructed two types of superlattices interface, namely LaO/TiO_2_ and BaO/AlO_2_ interfaces. To simulate the epitaxial film growth in the experiment, we fixed the lattice constants in the ab-plane, and fully relaxed all atoms (unless otherwise stated) in the LaAlO_3_/BaTiO_3_ perovskite superlattices.

## Electronic supplementary material

Supplementary Information
