# Supplementary material for: Thickness Control of the Spin-Polarized Two-Dimensional Electron Gas in LaAlO3/BaTiO3 Superlattices
Source: Sci Rep. 2018 Jan 11;8:467. doi: 10.1038/s41598-017-18858-x (PMC5765129; doi:10.1038/s41598-017-18858-x)
Supplement: Supplementary file 1 — Supplementary Information [file 41598_2017_18858_MOESM1_ESM.pdf]

Supplementary Information

Thickness Control of the Spin-Polarized Two-Dimensional Electron Gas in $LaAlO_3/BaTiO_3$ Superlattices

Chen Chen[1], Le Fang[2], Jihua Zhang[1,3], Guodong Zhao[1], and Wei Ren[1,2]

[1]International Centre for Quantum and Molecular Structures, Physics Department, Shanghai University, Shanghai 200444, China

[2]Materials Genome Institute and Shanghai Key Laboratory of High Temperature Superconductors, Shanghai University, Shanghai 200444, China

[3]Guizhou Provincial Key Laboratory of Computational Nano-Material Science, Guizhou Education University, Guiyang 550018, China

* renwei@shu.edu.cn

We have considered carefully the effect of ferroelectric $BaTiO_3$ on the $BaTiO_3/LaAlO_3$ superlattices. In detail, we constructed tetragonal $BaTiO_3$ with lattice constants a=b=3.9945Å and c=4.0335Å for the different $(LaAlO_3)_n/(BaTiO_3)_n$ superlattices (n=2.5 to 8.5). Then we relaxed these superlattices by fixing lattice constants a=b values. By observing the optimized structures, indeed a homogenrous ferroelectric polarization could not be found even for $(LaAlO_3)_{2.5}/(BaTiO_3)_{14.5}$ superlattice, with 6nm-thick $BaTiO_3$.

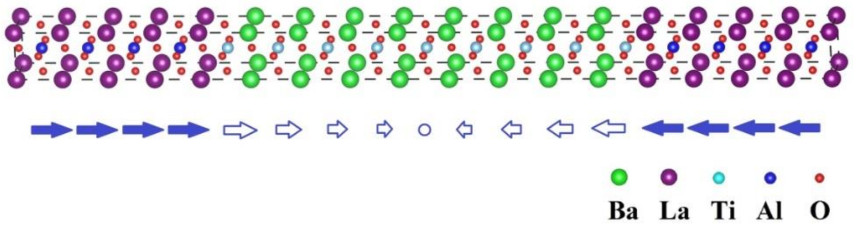

Figure S1. Atomic structures of the supercells with the LaO/TiO_2 interfaces in $(LaAlO_3)_{8.5}/(BaTiO_3)_{8.5}$ superlattice. The arrows represent the local dipole distribtuion of the corresponding atomic layers.

In our superlattices, near the two interfaces TiO_2/LaO, there should exist opposite polar modes pointing towards the bulk inside $BaTiO_3$. See Fig. S1 here please. In order to avoid the energy-consuming charged domain wall in the middle of $BaTiO_3$ the polarization diminishes.

Now we discuss the impact of "polar" effect in our $(LaAlO_3)_n/(BaTiO_3)_n$ superlattices, in comparison to the paraelectric superlattices. As we plot in Fig. S2 here the band structures of the $(LaAlO_3)_n/(BaTiO_3)_n$ superlattices with polar $BaTiO_3$, two separate bands are found near the conduction band minima which were actually almost degenerate in the exact mirror-symmetric $BaTiO_3$ case (see manuscript Fig. 6). The residual polarization induced band splitting is significantly as large as 0.45 eV. As a result of such band structure change, we would expect different behaviors for charge carrier density and transport properties under bias voltage.

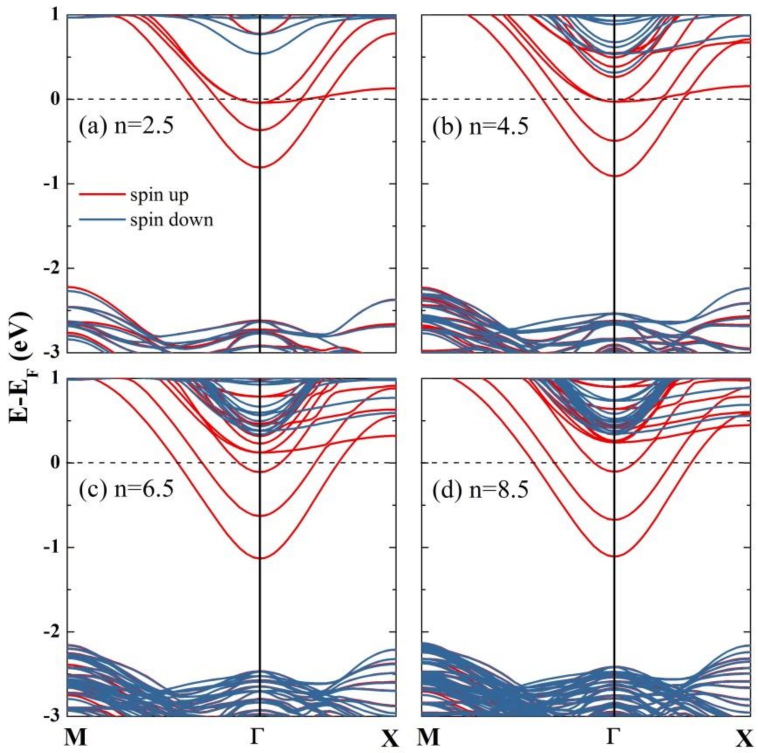

Figure S2. Electronic band structures of the $(LaAlO_3)_n/(BaTiO_3)_n$ superlattices (n=2.5, 4.5, 6.5, and 8.5) containing relaxed ferroelectric $BaTiO_3$ (and residual polarization) with two interfacial TiO_2 layers as shown in Fig. S1 above.

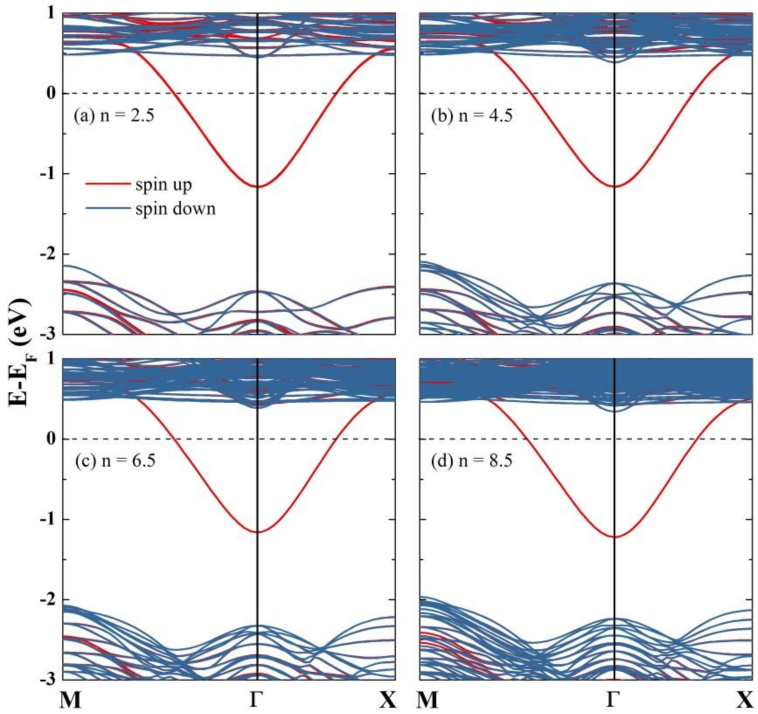

Figure S3. Electronic band structures of the highly symmetric non-polar $(LaAlO_3)_n/(BaTiO_3)_n$ (n=2.5, 4.5, 6.5, and 8.5) superlattices (no geometry relaxation is done).

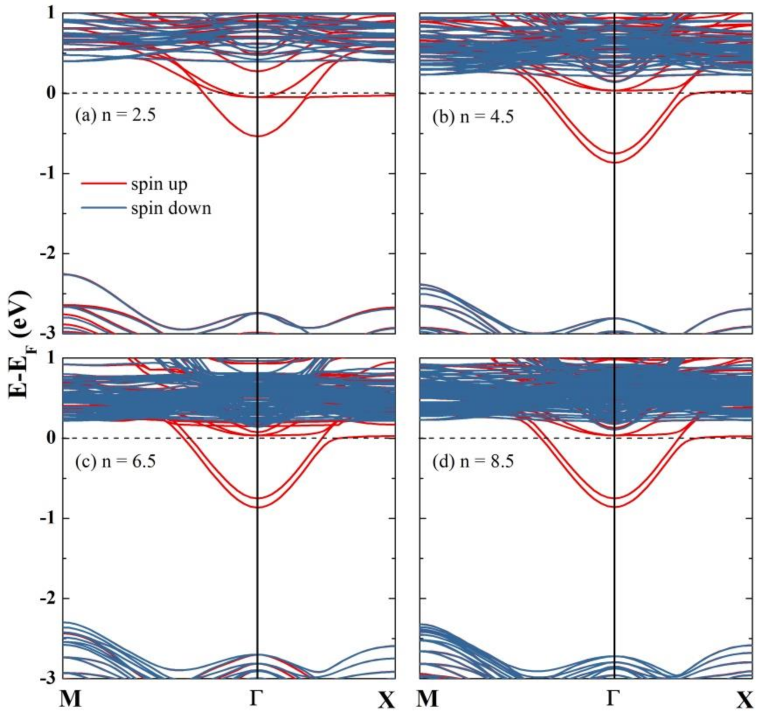

Figure S4. Electronic band structures of the polar $(LaAlO_3)_n/(BaTiO_3)_n$ superlattices (n=2.5, 4.5, 6.5, and 8.5) containing uniaxial polarized ferroelectric $BaTiO_3$ (no geometry relaxation is done).

In order to more intuitively understand the nature of the $(LaAlO_3)_n/(BaTiO_3)_n$ superlattices, we plotted ideally symmetric non-polar and ferroelectric polar (without any geometry relaxtion) superlattices in Fig. S3 and S4, respectively. We found that in the non-polar $(LaAlO_3)_n/(BaTiO_3)_n$ superlattices as shown in Fig. S3, the two mirror-symmetrical interfaces result in overlaped two-fold degenerate bands across the Fermi level. Their band minimum is about 1.2 eV below Fermi energy, quite independent of the parameter n. In Fig. S4, we assume uniaxially polarized ferroelectric $BaTiO_3$ exist in the $(LaAlO_3)_n/(BaTiO_3)_n$ superlattices. The two-fold degenerate bands are now splitted with an amplitude of 0.11 eV. Moreover, a flat band at the Fermi level is clearly observed. We speculate that such flat band is not stable and will be gone as we shown for the relaxed structure results in Fig. S2 above.

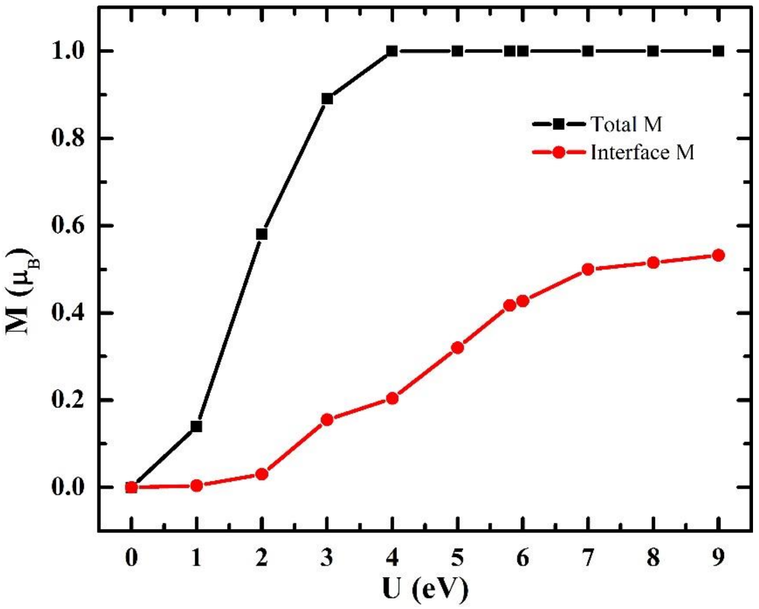

Figure S5. Calculated total magnetic moment and the magnetic moment of the Ti atom at the interface for the $(LaAlO_3)_{4.5}/(BaTiO_3)_{4.5}$ superlattice as a function of the Hubbard U value.

A more systematic study on the Hubbard U dependence has been done for the superlattice $(BaTiO_3)_{4.5}/(LaAlO_3)_{4.5}$, as shown in the following Figures S5 and S6. As can be seen from Figure S5, the total magnetic moment tends to be 1.0 μ_B at U = 4 eV,

whereas the magnetic moment of interfatial Ti atom increases monotonically with U value. As can be seen from the Figure S6, the interface charge density tends to be stable at U = 6 eV. By considering the total magnetic moment, the interface charge density, and references [Yang, K. S. et al, J. Phys. D: Appl. Phys. 47, 275101, (2014), Pentcheva, R. et al, Phys. Rev. B 78.205106 (2008)], we chose U = 5.8 eV for Ti 3d electrons.

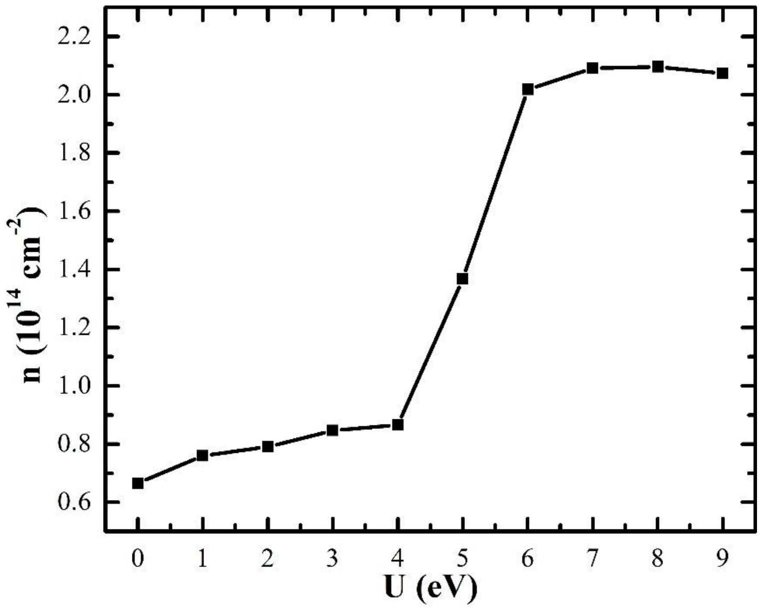

Figure S6. The calculated interface charge density for the $(LaAlO_3)_{4.5}/(BaTiO_3)_{4.5}$ superlattice as a function of the Hubbard U value.

Here, we constructed and calculated the superlattice $(SrTiO_3)_{4.5}/(LaAlO_3)_{4.5}$ by using the same parameters in our paper. The results show that the magnetic moment of the Ti atom at the interface is 0.32 μ_B, and the two-dimensional electron gas density at the interface is 1.48×10^{14} cm^{-2}. On the other hand, the magnetic moment of the Ti atom at the interface of the calculated superlattice $(BaTiO_3)_{4.5}/(LaAlO_3)_{4.5}$ is 0.42μ_B, and the density of the two-dimensional electron gas at the interface is 2.23×10^{14}cm^{-2}. The theoretical results indeed show that the $BaTiO_3/LaAlO_3$ superlattice has larger electron density than the conventional $SrTiO_3/LaAlO_3$ superlattice, though at the same order of magnitude. For $LaAlO_3/SrTiO_3$ superlattice we found that there is electron density penetrating into the $SrTiO_3$ near the interface. This effect was also clearly seen

in Ref. 15 of the manuscript. See Fig. 2 of *J. Appl. Phys.* **117**, 115305, (2015). This explains why we could obtain a higher electron density of $LaAlO_3/BaTiO_3$ superlattice than that of $LaAlO_3/SrTiO_3$ superlattice. We plot the layer-resolved partial DOS for $(SrTiO_3)_{4.5}/(LaAlO_3)_{4.5}$ superlattice, as shown in the Figure S7.

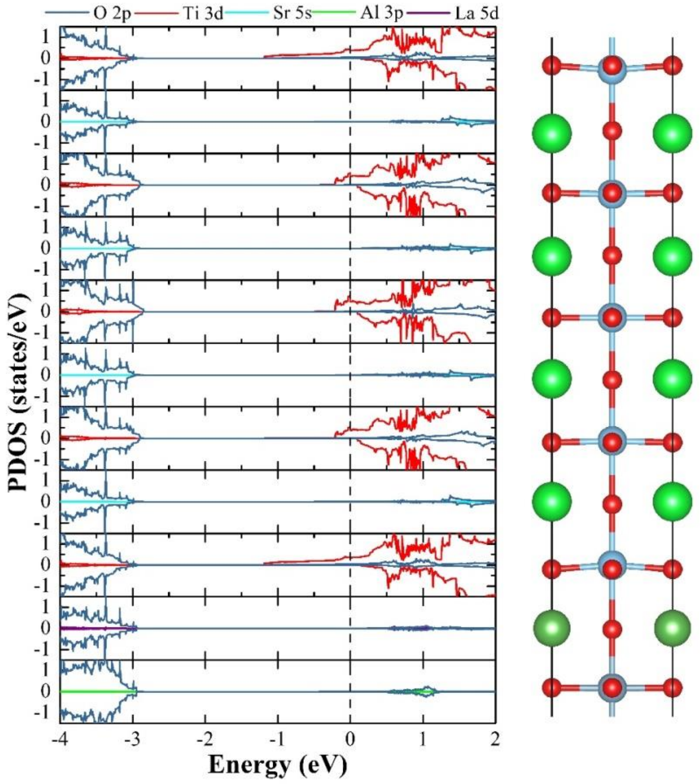

Figure S7. Calculated layer-resolved partial DOS of the interfacial TiO_2 monolayer for the $(SrTiO_3)_{4.5}/(LaAlO_3)_{4.5}$ superlattice in the range from -4.0 to 2.0 eV.
